# HIV/AIDS Epidemic and COVID-19 Pandemic in Africa

**DOI:** 10.3389/fgene.2021.670511

**Published:** 2021-08-31

**Authors:** Abdullahi Tunde Aborode, Athanasios Alexiou, Shoaib Ahmad, Mohammad Yasir Essar, Osuji Samuel Chibueze, Yahea Al-Zahrani, Oni-Ebenezer Ayomide, Gaber El-Saber Batiha

**Affiliations:** ^1^Department of Chemistry, University of Ilorin, Ilorin, Nigeria; ^2^Novel Global Community Educational Foundation, Hebersham, NSW, Australia; ^3^AFNP Med Austria, Wien, Austria; ^4^Punjab Medical College, Faisalabad, Pakistan; ^5^Medical Research Center, Kateb University, Kabul, Afghanistan; ^6^Department of Optometry, Madonna University, Okija, Nigeria; ^7^Department of Public Health, Federal University of Technology, Akure, Nigeria; ^8^Department of Internal Medicine, College of Medicine, Taif University, Taif, Saudi Arabia; ^9^Department of Biochemistry, Faculty of Life Science, Adekunle Ajasin University, Ondo, Nigeria; ^10^Department of Pharmacology and Therapeutics, Faculty of Veterinary Medicine, Damanhour University, Damanhour, Egypt

**Keywords:** COVID-19 pandemic, HIV epidemic, Africa, public health system, intervention

## Introduction

COVID-19 pandemic has transmitted significantly and become ubiquitous globally, instantly as the disease's information spread from a city in China named Wuhan since December 2019, it become a global public health threat. COVID-19 was declared a pandemic on March 12, 2020, by the World Health Organization (Huang et al., 2020). Research has shown that about 37.9 million people who are HIV carriers (Joint United Nations Programme on HIV/AIDS, 2020) are vulnerable to severe acute respiratory syndrome coronavirus-2 (SARS-CoV-2), which results in Coronavirus Disease 2019 (Joint United Nations Programme on HIV/AIDS, 2020).

Various African countries have been responding to different health problems such as HIV/AIDS and Tuberculosis, which indicates that millions of people are immune-compromised and many are vulnerable to high health problems due to the respiratory disorder obtained from the virus. In the same vein, the increased number of people having malaria in African countries has made more people susceptible to it (Aborode et al., 2021a). This may deceive diagnostic testing because high fever is part of the symptoms of both malaria and COVID-19 (Aborode et al., 2021a).

The inception of a new disease can have a catastrophic and long-lasting effect on already fragile health systems. For instance, about 10,600 people lost their lives to HIV/AIDS, Malaria, and Tuberculosis in some countries in West Africa due to the weakened healthcare system caused by the Ebola epidemic (Aborode et al., 2021b). Painfully, parents' and children's health was highly reduced, and no iota of recovery was recorded after the epidemic. Another example is the Republic of Congo, where the health sector lost its focus on reducing measles transmission due to the outbreak of Ebola in some parts of the country (Yoo, 2020). Although various international organizations are in partnership with the governments and local collaborators in improving services to HIV carriers, the COVID-19 pandemic outbreak has led to different challenges in implementing these services (McCloskey et al., 2014).

However, the introduction of quarantine, social distancing, and self-isolation strategies has led to people's inability to access continued HIV testing. As a result, this might affect the completion of UNAIDS's first 90-90-90 global target (Levi et al., 2016). Secondly, the time plan organized for HIV/AIDS care service could be affected during the COVID-19 pandemic. HIV carriers might be delayed in getting antiretroviral therapy (ART) in hospitals because hospitals' focus is to treat patients with COVID-19 pandemic and Healthcare visitation has been restricted due to the country's implementation lockdowns (Jiang et al., 2020; World Health Organization, 2020b). Also, to control COVID-19 pandemic transmission and vaccine production, most public health funds are targeted at organizations focused on achieving these goals, thereby reducing the resources allocated for HIV/AIDS care services (Von Bogdandy and Villarreal, 2020).

## Pandemics Should Reform Africa's Public Health System

Some recent scientific studies have looked into the connections between HIV/AIDS and COVID-19 pandemic, and few similarities were reported. For instance, research carried out by He et al. provided evidence that COVID-19 pandemic reduces T-Lymphocytes which is similar to the mechanism of HIV (He et al., 2020). Another research by Guillen et al. reported that different individuals with COVID-19 pandemic severe cases might have lymphopenia or an atypically low number of lymphocytes in the blood (Guillen et al., 2020).

Another significant similarity between HIV/AIDS and the novel coronavirus disease 2019 (COVID-19) is that there are no licensed pharmaceuticals for COVID-19 vaccine or drug research, just as during the early days of the HIV/AIDS pandemic. As a result, people's behavior toward the pandemics will determine the pandemic trajectory of COVID-19 (Anderson et al., 2020), just as it is for HIV/AIDS.

However, COVID-19 and HIV/AIDS exhibit some differences too. Firstly, untreated and unattended HIV/AIDS infection usually leads to the patient's death, while COVID-19 pandemic most times kills people with underlying health conditions and old age. Secondly, behavioral changes expected to reduce the rate of transmission are different. For HIV/AIDS, reducing sexual behavior and needle sharing is very important for COVID-19, physical proximity, and handwashing (May and Anderson, 1988).

Besides, the time interval for the infections is different. For HIV/AIDS, early cases increased over 6–12 months, while for COVID-19 pandemic, the interval of infection is a matter of days. In light of these difficulties in managing COVID-19 with HIV/AIDS, WHO, UNAIDS, and the Global Network of People Living with HIV are cooperating to improve with the arrangement of HIV anticipation, testing, and treatment administrations (UNAIDS, 2020a,b; World Health Organization, 2020c). On March 20, 2020, The US Department of Health and Human Services discharged effective methods for COVID-19 and HIV/AIDS carriers (UNAIDS, 2020b), which guaranteed that HIV/AIDS patients ought to keep up at least a 30-day supply and preferably a 90-day supply of ART and all other medications if possible.

In the same vein, the Chinese National Center for AIDS/STD Control and Prevention certified information guaranteeing free antiviral drugs for special treatment management agencies in China and released a list of ART clinics (UNAIDS, 2020a). HIV/AIDS carriers can retake antiviral drugs either at the nearest local center for Disease Control and Prevention or by post to maintain enrolment in treatment programs and continue ART (UNAIDS, 2020a). Furthermore, healthcare suppliers in Thailand are to administer antiviral medications in 3–6-month dosages to meet HIV/AIDS bearers' therapeutic requirements and decrease office visits. Besides, community-based organizations are playing significant roles in keeping up HIV administrations. For example, UNAIDS is associated with the BaiHuaLin coalition of HIV/AIDS carriers and other organizations to reach and help the individuals who will come up short on antiviral medications soon (UNAIDS, 2020a).

Since Wuhan's lockdown on January 23, 2020, a network-based association (Wuhan Tong Zhi Center) has committed assets to guarantee the supply of antiviral medications and opened a hotline to provide consultations. As of March 31, 2020, this association has provided 5,500 counsels with individuals living with HIV and has helped about 2,664 people get antiviral medications. The Thai Red Cross AIDS Research Center set up visible platforms outside their facility with a screening framework for people, giving HIV testing and avoidance supplies (e.g., condoms, post-exposure prophylaxis, and pre-introduction prophylaxis) (UNAIDS, 2020b). As COVID-19 keeps on spreading worldwide, many developing countries face the danger of SARS-CoV-2 disease with hindrances and difficulties in keeping up the HIV care continuum. The circumstance could be more terrible in places with frail healthcare services frameworks. For example, in Nigeria, as revealed in a Feature by Paul Adepoju, the danger of SARS-CoV-2 affects HIV and tuberculosis reactions as patients decide to socially separate by not going to their health providers for treatment and drugs collection (Adepoju, 2020). The reactions to COVID-19 in low-resources, high HIV burden settings will fundamentally be different from the high-asset settings to a great extent.

Some countries have moved to strict movement controls, recognizing that the informal sector provides jobs for the vast majority of citizens. In sub-Saharan Africa, frameworks set up to manage HIV and the cleverness that portrays the healthcare services reaction may be an incredible resource in the battle against the new pandemic (De Cock et al., 2003).

Furthermore, the involvement with battling for reasonable access to new medicines might be more significant than any other time in the coming weeks. For the time being, SARS-CoV-2 will distract the attention given to HIV, disrupt treatment and prevention programs, and may lead to a rise in disease burden and even HIV incidence as a result. Several pieces of research suggest that severe interruption of antiretroviral therapy services during COVID-19 could lead to a 1.5- to 3-fold increase in mortality (Adepoju, 2020; UNAIDS, 2020b; World Health Organization, 2020c). Like HIV, the spread of the COVID-19, which as of June 4, 2020, had infected more than 6,151,298 individuals globally and caused 388,459 deaths, which is joined by stigma (World Health Organization, 2020a).

Around the globe, stigmatizing conduct is accounted for against those diagnosed with COVID-19 and individuals are seen as conceivably contaminated with the coronavirus regularly because of their national starting point (Al Jazeera, 2020). For instance, In the Central African Republic, the declaration of the first COVID-19 constructive individual, a Catholic minister who had lived in the nation for a long time and had quite recently come back from an outing to Italy, prompted verbal and composed assaults against the patient and Catholics and outsiders by and large viewed as vectors of the illness (Radio Ndeke Luka, 2020). As COVID-19 keeps on spreading far and wide, so too have bits of rumors, misinformation, and fake news about the pandemic. Recordings, voice messages, writings, and stories have swirled around clashing data, from problematic fixes to strange cases that Africans are in one way or another safe from COVID-19, regardless of an abundance of opposite proof.

Tending to the damages of falsehood should, therefore, be a priority with COVID-19, and indeed compelling reactions to the pandemic would incredibly benefit from all the exercises of the multi-sectoral and rights-based ways to deal with the HIV epidemic. The impact of COVID-19 on HIV state in Africa particularly in weak healthcare systems will become double burden considering the insufficient medical resources and weak diseases surveillance will disrupt the attention of HIV patients to have easy access to healthcare centers, seek medical attention and increase drug shortage such as ART drugs for HIV patients (Nachega et al., 2021). The disproportionate proportion of COVID-19 pandemic on HIV and other vulnerable people like children, old aged, sickle cell diseases, and undernutrition is a significant burden for the underequipped healthcare systems in Africa to fight and contain and this, therefore, increase the mortality and morbidity rates of infections in Africa and affect child health (Coker et al., 2021).

## Efforts and Recommendations

Viable public health responses must be grounded in sound logical proof on the methods of transmission of the epidemic, its prevention, and (potential) treatment. Logical proof must guide the activities of political pioneers and chiefs. Health experts and health institutions upheld by the World Health Organization (WHO) assume a fundamental job in the turn of events and dispersal of logical information on the epidemic and reaction. Proof on the prevention and management of COVID-19 must be very much conveyed to the media and networks, with unique endeavors to address “counterfeit news” and expose myths.

There is need for Africa healthcare systems should increase and improving their diseases testing among priority and undertested and underprivileged populations during the pandemic. There should be a strategic plan organized by national and international technical organizations that will create an avenue where populations can test for HIV by distributing the HIV kits and create awareness and education on how to test, while COVID-19 testing should be made available everywhere where anyone can have access anytime.

Positive encounters from nations confronting the epidemic should manage reactions elsewhere. In the battle against HIV, encounters from Senegal, Thailand, Switzerland, and Uganda were methodically portrayed and utilized as outstanding practice. Regarding COVID-19, encounters from China and South Korea are being utilized, and bits of knowledge from early triumphs should be made promptly accessible (Al Jazeera, 2020). Advancements have likewise been executed to help those families generally vulnerable to mobility restrictions and the economic hardships this creates. Helping them meet fundamental work needs, for example, access to food, can lessen the danger of spread in Africa's many rambling urban informal settlements where COVID-19 could spread like wildfire because of a big clog, poor cleanliness, and previous well-being conditions.

Given Africa's significant destitution levels, lockdowns without social security plans could prompt serious outcomes, including starvation and consumption of ways of dealing with stress, especially among the most powerless. Conflicts between residents and security forces resulting from movement restrictions have prompted deaths and wounds in Nigeria, Rwanda, South Africa, and Uganda (Crisis Group, 2020). Helping them meet basic livelihood needs, for example, access to food, can diminish the danger in Kenya's casual settlements. Indigenous associations, for example, Mutual Aid Kenya and various associations are strengthening the administration's reaction system by identifying at-risk families and providing targeted assistance through direct cash transfers, food bundles, and elective supply chains to give essential items spread in Africa's many rambling urban casual repayments where COVID-19 could fan out quickly because of gigantic blockage, poor cleanliness, and previous well-being conditions (Radio Ndeke Luka, 2020).

In Kibera, the biggest of these settlements, a network-run association called Shofco has set up handwashing stations, network toilets, and clean-water booths in all passageways, staffed by volunteers and a system of health workers. Three thousand of the territory's most helpless families accept an immediate money move of $24 every month for 3 months to meet their fundamental needs, with financing originating from the private neighborhood segment and the Kenya Diaspora in North America (Duerksen, 2020). In Botswana, a paid sponsorship totaling 1 billion pula ($84 million) has been given to independent companies as a motivation to hold their employees during the shutdown. The administration will also contribute 50 percent of the basic pay of each furloughed resident or perpetual occupant for 3 months, alongside the sponsorship of 1,000–2,000 pula ($80–168) every month to address fundamental issues (Africa Center for Strategic Studies, 2020; Duerksen, 2020; Smith, 2020).

Africans are responding to the challenge in various manners. In South Africa, a private firm, Praekelt.org, made a WhatsApp-based helpline that gives continuous information and robotized reactions in various dialects to teach and sharpen. The application enlisted 3.5 million endorsers inside the initial 10 days of launching. Praekelt.org has now cooperated with the World Health Organization to do a similar service to reach a global audience.

Africa Check, Africa's pioneer fact-checking association, gives devoted COVID-19 assistance in an organization with Facebook. Facebook is additionally working with Nigerian media organizations to battle falsehood via web-based networking media. The Nigerian Presidential COVID-19 Task Force has likewise established a 24-h hotline giving forward-thinking data to educate and shield the general population from deception and bits of gossip. Numerous presidents and senior well-being pioneers utilize their day-by-day briefings to dispel bits of gossip and deception about COVID-19 (Africa Center for Strategic Studies, 2020; Duerksen, 2020; Smith, 2020).

We must think to rebuild and reshape the HIV response once the initial wave of COVID-19 is passed and nations learn to live with the dual pandemic. Like HIV, the COVID-19 pandemic isn't just a well-being concern, but it is a social, economic, and human security issue. However, as part of COVID-19 pandemic preparedness, maintaining a sufficient supply of ART is critical. A public health response is a need in establishing and maintaining a consistent drug supply chain. The United Nations Security Council perceived HIV as a harmony and security issue on January 10, 2000, when it met to examine the scourge's effect in Africa. This was the first run through the Security Council, which had tended to a medical problem as a danger to harmony and security, making ready for the appropriation of Resolution 1,308 on HIV/AIDS and worldwide peacekeeping tasks (UN Security Council, 2000).

Reacting to pandemics, for example, HIV and COVID-19, require a multi-sectoral approach that activates initiative at the most significant level. From Malaysia to Uruguay, to Italy and the Central African Republic, Heads of State and governments are occupied with the reaction to COVID-19 and are administering measures to control its spread. The inclusion of Heads of State is expected to bring all offices and organizations into the reaction, initiate emergency instruments and assets, and pass on the circumstance's direness.HIV pandemic is a significant cause of multilateralism and worldwide cooperation. Thanks to network activism, global solidarity, and collaboration in the fields of science and medication, 24.5 million individuals are on antiretroviral treatment today, generally in poor and middle-income nations (UNAIDS, 2019).

The United Nations Secretary-General and the Director-General of WHO at the G20 Leaders' Extraordinary Summit on COVID-19 on March 26, 2020, focused on the critical need to quicken worldwide organization solidarity in response to the pandemic (United Nations, 2020b). This solidarity must be tied down in a multilateral system to help and finance the worldwide reaction and recuperation with explicit regard for nations generally influenced and those generally delicate. These standards are additionally explained in the Secretary General's report, Shared duty, worldwide solidarity: Responding to the economic effects of COVID-19 (United Nations, 2020a).

With a health system severely debilitated by many years of political precariousness and strife, perhaps the most negligible proportion of qualified well-being laborers per capita on the planet, and the more significant part of its populace needing helpful help, the Central African Republic is one of the most delicate nations confronting COVID-19 (Coordination of Humanitarian Affairs, 2020). With the help of WHO, MINUSCA, the World Bank, UNICEF, and other UN offices and accomplices, early measures adopted by the legislature seem to have been compelling with just six essentially imported instances of COVID-19 recorded toward the end of March and constrained proof of nearby transmission.

As nations adopt various strategies to control the pandemic, we should portray what measures are working by and assess how individuals react, and be aware of unintended impacts. Similarly, modelers must shield their forecasts, so policymakers should clarify their behavioral interventions' proof and hypothesis. Straightforwardness encourages assessment and empowers examining suspicions, prompts better practice, and tackles thoughts from a scope of logical orders.

## Conclusion

As COVID-19 keeps on spreading the world over, numerous areas are confronting the danger of SARS-CoV-2 disease and obstructions and difficulties for keeping up the HIV care on high standards. The situation, unfortunately, is worse in countries with weak healthcare systems. We suggest that legislatures, network-based associations, and international accomplices should cooperate to keep up the HIV care continuum during the COVID-19 pandemic, with specific endeavors to guarantee convenient access to and maintain a strategic distance from disturbance routine HIV administrations.

## Author Contributions

ATA conceptualized the research idea. AA and GB review and make substantial revision on the first draft. ATA and GB write the second draft and AA revised the second draft. All authors draft out the first manuscript. All authors read and revise and approve the final draft of the manuscript.

## Conflict of Interest

The authors declare that the research was conducted in the absence of any commercial or financial relationships that could be construed as a potential conflict of interest.

## Publisher's Note

All claims expressed in this article are solely those of the authors and do not necessarily represent those of their affiliated organizations, or those of the publisher, the editors and the reviewers. Any product that may be evaluated in this article, or claim that may be made by its manufacturer, is not guaranteed or endorsed by the publisher.
